# Sex-Linked Differences in Pulmonary Functions of COVID-19 Patients After a Six-Minute Walk Test

**DOI:** 10.7759/cureus.50071

**Published:** 2023-12-06

**Authors:** Syed S Raza, Umema Zafar, Dur E Shehwar, Hamna Zafar, Farhan Ullah, Maha Wazir, Syed Muhammad H Abbas, Hina Wazir, Hunya Amin, Giustino Varrassi

**Affiliations:** 1 Physiology, Khyber Medical College, Peshawar, PAK; 2 Medicine, Khyber Medical College, Peshawar, PAK; 3 Internal Medicine, Khyber Medical College, Peshawar, PAK; 4 Pain Medicine, Paolo Procacci Foundation, Rome, ITA

**Keywords:** six-minute walk test, spirometry, pulmonary functions, stress, gender comparison, sex differences, covid-19

## Abstract

Coronavirus disease 2019 (COVID-19) predominantly impacts the respiratory system. Historically, numerous lung diseases have shown sex-related differences throughout their progression. This study aimed to identify sex-linked disparities in pulmonary function tests (PFTs) among individuals who have recovered from COVID-19 when subjected to a six-minute walk test (6MWT). In this observational cross-sectional study, we analyzed 61 participants, consisting of 39 (64%) males and 22 (36%) females, all of whom previously contracted COVID-19 three months or more prior. We measured vitals such as blood pressure, pulse, oxygen saturation, and PFT values before and after the 6MWT. The post-6MWT evaluation revealed notable mean differences between males and females in parameters systolic blood pressure (SBP) (p = 0.003), diastolic blood pressure (DBP) (p = 0.026), forced expiratory volume in the first second (FEV1) (p = 0.038), forced vital capacity (FVC) (p = 0.041), and maximum voluntary ventilation (MVV) index (p = 0.011). PFT outcomes indicated sex-based variations among post-COVID-19 subjects. Specifically, post-stress values for FEV1, FVC, MVV index, SBP, and DBP were more elevated in males than in females. However, females presented with higher oxygen saturation levels post-COVID-19 compared to males. Using multiple linear regression modeling, sex was not found to be a strong predictor of PFT results. However, individual regression analyses for FEV1, FVC, and MVV index consistently showcased higher values in males. In conclusion, significant PFT differences exist between males and females after recovery from COVID-19 when exposed to stress induction via the 6MWT.

## Introduction

Coronavirus disease 2019 (COVID-19) is caused by severe acute respiratory syndrome coronavirus 2 (SARS-CoV-2). It has globally affected millions of people in general and has had debilitating effects on lung functions in particular [1]. Males have larger lungs than females [2,3], and the mean values of pulmonary variables are also significantly higher for males than for females [4,5]. These sex-based anatomical and physiological differences in the respiratory system become critically important during exercise and disease [6]. COVID-19 infection affects the respiratory system, and studies have found that a sex-related difference exists in many lung diseases throughout the life span [7,8]. Multiple studies have suggested a complex interplay of genetics, sex hormones, host immunity, anatomical and physiological differences, and sociocultural and behavioral factors that are likely to underlie the observed sex differences in infection rates and their severity [9-12]. Females have smaller chest diameters, and the trachea and bronchioles also have smaller diameters. Lung volumes, capacities, and flow rates are also smaller in females compared to males. LoMauro et al. believe that the size of air passages, rather than hormonal differences between genders, is responsible for gender-based differences in baseline pulmonary function tests (PFTs) [6].

All infectious diseases elicit an inflammatory response in the affected organ. Various reports have revealed prolonged lung function impairment in post-COVID-19 patients [13-15]. The literature suggests that males exhibit an exaggerated inflammatory response to external agents such as bacteria, viruses, or fungi. This overwhelming inflammatory response is mediated by pathogen recognition receptors (PRRs). These are also known as Toll-like receptors. Gender differences in these TLRs indicate that inflammation is more likely to be caused by infectious agents rather than non-infectious factors [11]. Variations have been observed in how males and females respond to different respiratory infections. The altered respiratory function, secondary to infection, raises concerns about long-term respiratory complications and physical performance in COVID-19-affected individuals [15].

The American Thoracic Society (ATS) introduced a six-minute walk test (6MWT) along with comprehensive guidelines [16]. This sub-maximal exercise test is used to measure aerobic capacity and strength. The 6MWT can be used in all age groups, ranging from kids to adults, and for a wide range of diagnoses. This test is designed to assess patients with cardiopulmonary issues and provides an objective evaluation and important information about the body systems during the period of physical activity, including the body’s metabolism, circulatory system, cardiovascular system, pulmonary system, and neuromuscular units [17].

PFTs are a diagnostic tool used to assess lung function. Mo et al. checked the PFTs of COVID-19 patients at the time of discharge and found the following abnormalities in the study participants: diffusion capacity was abnormal in 47.2% of the patients, total lung capacity (TLC) in 25%, forced expiratory volume in the first second (FEV1) in 13.6%, forced vital capacity (FVC) in 9.1%, and FEV1/FVC in 4.5% [13].

At present, however, the evidence on the impact of sex-linked differences in pulmonary functions of COVID-19 patients after a six-minute walk test remains poor, and no accurate study is available regarding sex-linked differences in the pulmonary functions after stress induction. In this research, our aim was to assess sex-linked differences in pulmonary function tests (PFTs) of COVID-19 patients after a six-minute walk test (6MWT) three months post-COVID-19 infection.

## Materials and methods

Subjects and procedure

**Figure 1:**
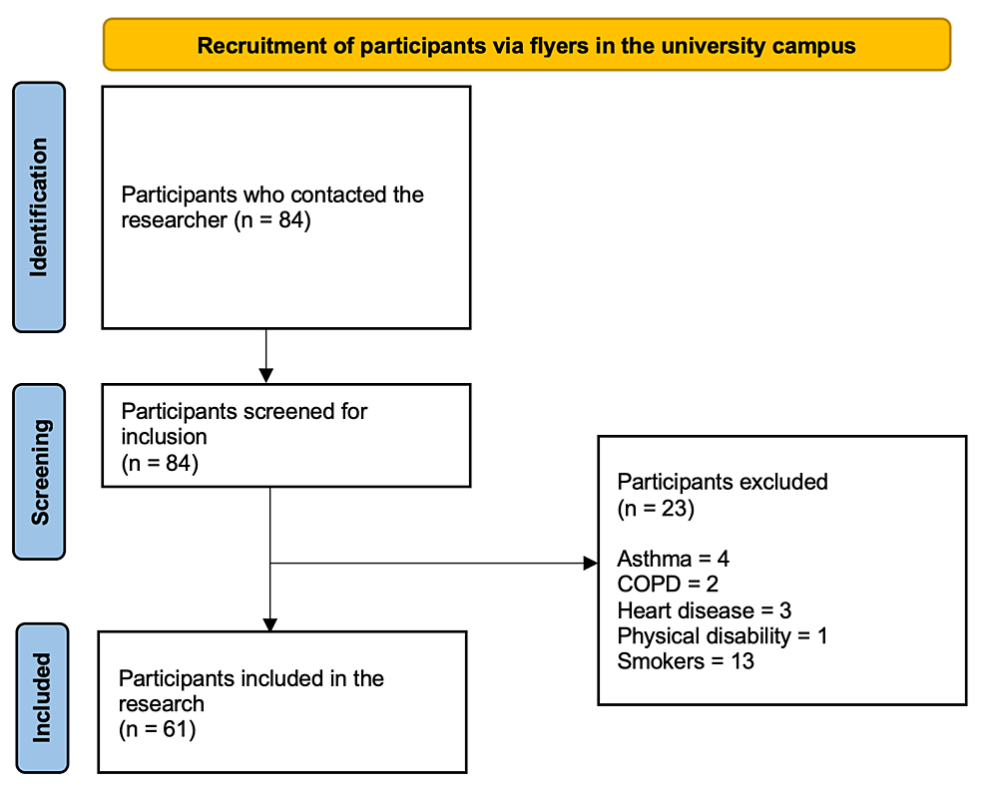
Recruitment of participants COPD: chronic obstructive pulmonary disease

This study was conducted at Khyber Medical College in the Department of Physiology from October 2021 to December 2021. Approval for the study was granted by the Ethical Review Board of Khyber Medical College (approval numbers: 828/DME/KMC and 07-02-2021). This is an observational cross-sectional study consisting of 61 participants. The sample size was determined using G*Power. The participants were enrolled at the main campus of Khyber Medical University (KMU). Flyers containing contact details of researchers and a preliminary information sheet were distributed on campus. Those participants who responded were then screened based on inclusion and exclusion criteria. Participants aged 18-40 years of both genders who had previously contracted COVID-19 (three months or more had passed) were included. Their previous infection was confirmed through a polymerase chain reaction (PCR) test, and they had obvious symptoms that required hospitalization. Those individuals with respiratory diseases such as asthma, chronic obstructive pulmonary disease (COPD), heart disease, uncontrolled hypertension, and physical disabilities were excluded. Professional athletes and smokers were also excluded (Figure 1). The participants were provided with an information sheet. If they were not literate, they were verbally informed about the perspective, benefits, and risks of the study. The participants signed a written informed consent before commencing the study-specific screening.

All subjects recruited for the study were initially registered. An individual-specific registration number was assigned to each participant. The investigator responsible had to both verify the participant’s eligibility and obtain a signed informed consent/registration form.

PFTs and vitals, including blood pressure (BP), pulse, and oxygen saturation (the last two were recorded using a pulse oximeter), were measured after conducting a six-minute walk test in accordance with the ATS guidelines [16]. The participant was seated comfortably in a chair. The right arm was exposed to attach the cuff. The Omron M3 Basic blood pressure monitor (OMRON, Kyoto, Japan) was switched on, and BP was recorded immediately after the stress. The participants’ PFTs were then measured using a Vitalograph (spirometer). A disposable mouthpiece was provided to each participant. They were instructed to take a slow breath and exhale into the mouthpiece. Then, they were asked to take a deep breath and exhale forcefully into the mouthpiece of the Vitalograph (Spiron lab III Vitalograph) for the pulmonary function test.

Statistical analysis

After applying the tests of normality and setting the significance level at a p-value of 0.05 or less, the two-tailed unpaired t-test was applied. To assess the linearity of the relationship between variables, a matrix scatterplot was generated using the Statistical Package for the Social Sciences (SPSS) for MacBook Pro version 26.0 (IBM SPSS Statistics, Armonk, NY). Based on the scatterplot, the variables were checked for multicollinearity. To cross-check, a Pearson correlation table was created for all the variables.

Multiple linear regression was applied using the stepwise method. After analysis, the variables were excluded. In the next step, regression was applied using the forward method, and once again, the variables were excluded. Next, regression analysis was performed using the enter and remove method combined. In the enter method, all the variables were included. While in the remove method, the excluded variables were selected as independent variables.

## Results

A total of 39 (64%) males and 22 (36%) females were included in this study. The average age of males was 27 ± 6 years, while the average age of females was 26 ± 5 years. The average height of males was 169.87 ± 6.47 cm, while for females, it was 159.88 ± 3.30 cm. Similarly, for weight, males had a mean and standard deviation (SD) of 72.94 ± 13.72 kg, while females had 62.50 ± 8.37 kg. All these parameters were significantly different from each other (p < 0.05) (Table 1). The average time passed after COVID-19 infection for these individuals was 7.09 ± 2.86 months. Three (4.9%) cases had known comorbidity: one had type 2 diabetes mellitus and two had hypertension. The top three symptoms reported during the bout of COVID-19 attack in the participants were fever (86.9%), dry cough (73.8%), and body aches (90.2%). In this study, 72.1% of the participants consulted a physician for their respiratory illness. Additionally, 93.4% of the cases received treatment either after consulting with their primary physician or through over-the-counter self-medication. It is worth noting that a majority of cases were either doctors or medical students. Out of the total study participants (N = 61), only one participant needed hospitalization (Table 2).

**Table 1:** Demographic characteristics

Characteristic	Gender	Mean ± standard deviation	Mean difference	p-value
Age (years)	Male (n = 39)	27 ± 6	-1.0	0.002
	Female (n = 22)	26 ± 5		
Height (cm)	Male	169.87 ± 6.47	-9.99	0.000
	Female	159.88 ± 3.30		
Weight (kg)	Male	72.94 ± 13.72	-10.44	0.001
	Female	62.50 ± 8.37		

**Table 2:** Clinical characteristics COVID-19: coronavirus disease 2019

Characteristic	Number (value)
Comorbid	3 participants
Mean elapsed time (post-COVID-19)	7.09 ± 2.86 months
Top reported symptom: body aches	55 participants
Physician consulted	44 participants
Hospitalization	1 participant
Received treatment	57 participants

A comparison of means was conducted to analyze various parameters between post-COVID-19 males and females after performing the 6MWT. The results showed a significant difference in systolic blood pressure (SBP) (p = 0.003), diastolic blood pressure (DBP) (p = 0.026), FEV1 (p = 0.038), FVC (p = 0.041), and maximum voluntary ventilation (MVV) index (p = 0.011) (Table 3).

**Table 3:** Comparison of parameters between post-COVID-19 males and females after performing a six-minute walk test SBP: systolic blood pressure, DBP: diastolic blood pressure, HR: heart rate, VC: vital capacity, FEV1: forced expiratory volume in the first second, FVC: forced vital capacity, FEV%: forced expiratory volume %, FEF25: forced expiratory flow 25, FEF50: forced expiratory flow 50, FEF75: forced expiratory flow 75, MVV IND: maximum ventilatory volume index, PEF: peak expiratory flow

Variables	Gender	Mean ± standard deviation	Mean difference	Significance (two-tailed)	95% confidence interval of the mean difference
Female	98.81 ± 0.50				
SBP (mmHg)	Male	127.51 ± 13.79	-11.96	0.003	-19.64, -4.29
	Female	115.54 ± 15.39			
DBP (mmHg)	Male	88.35 ± 15.45	-8.35	0.026	-15.67, -1.04
	Female	80.00 ± 9.79			
HR (beats/minute)	Male	94.84 ± 16.42	4.47	0.363	-5.28, 14.23
	Female	99.31 ± 21.26			
VC	Male	103.94 ± 31.70	-0.18	0.985	-19.08, 18.72
	Female	103.76 ± 33.64			
FEV1	Male	120.61 ± 28.05	-17.11	0.038	-33.26, -0.96
	Female	103.50 ± 33.91			
FVC	Male	114.87 ± 27.69	-16.14	0.041	-31.60, -0.68
	Female	98.72 ± 31.14			
FEV%	Male	107.86 ± 6.80	0.742	0.676	-2.79, 4.28
	Female	108.60 ± 6.31			
FEF25	Male	112.05 ± 41.08	-17.91	0.122	-40.76, 4.93
	Female	94.13 ± 45.79			
FEF50	Male	110.28 ± 34.49	-15.50	0.127	-35.55, 4.53
	Female	94.77 ± 42.58			
FEF75	Male	116.72 ± 27.47	-4.57	0.56	-20.10, 10.95
	Female	112.15 ± 28.32			
MVV IND	Male	119.93 ± 39.09	-30.77	0.011	-54.22, -7.33
	Female	89.15 ± 51.58			
PEF	Male	108.46 ± 40.24	-20.87	0.061	-42.77, 1.03
	Female	87.59 ± 42.45			

**Figure 2:**
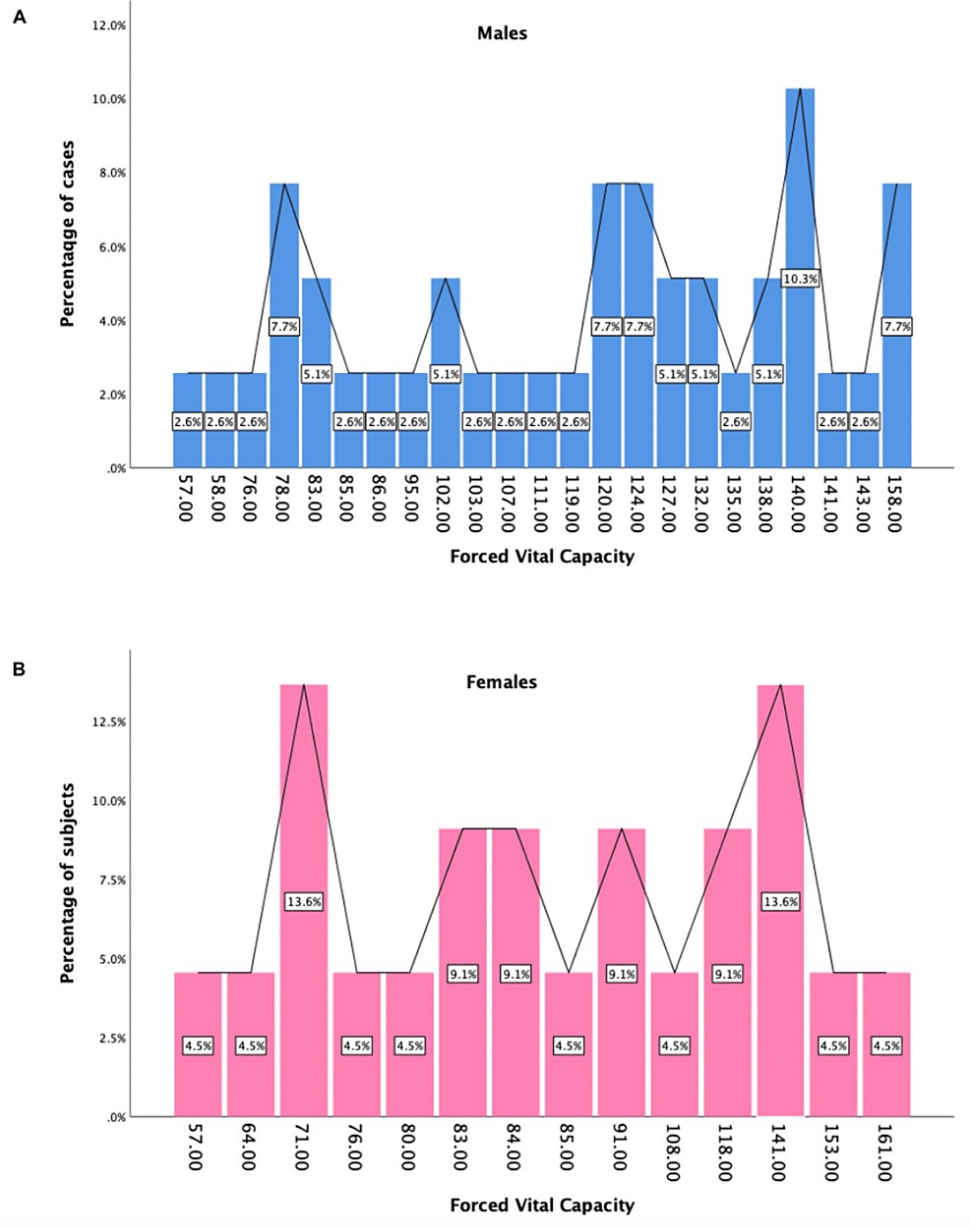
Sex-linked differences in the post-6MWT values of forced vital capacity A: forced vital capacity for males (blue graph), B: forced vital capacity for females (pink graph)

**Figure 3:**
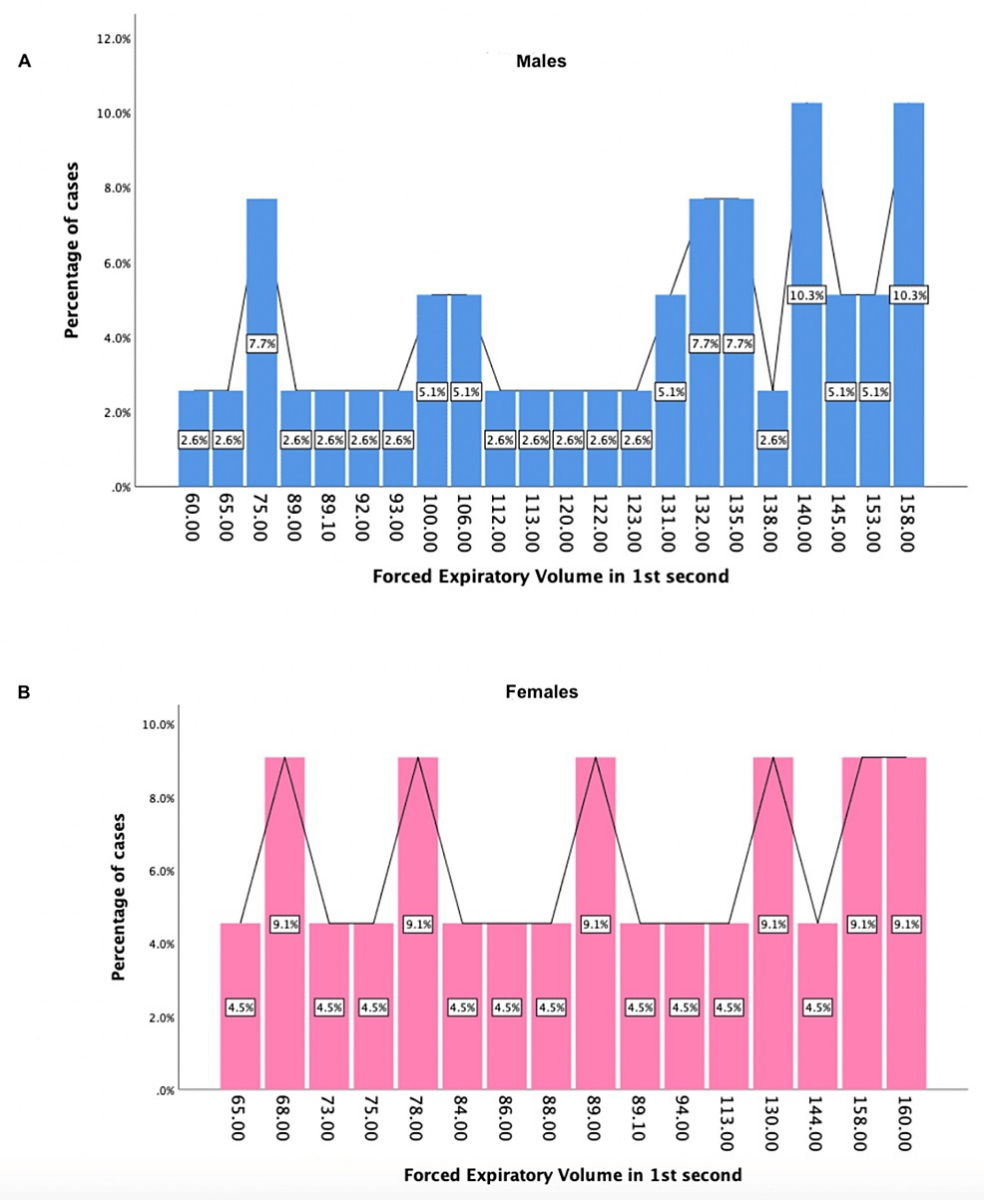
Sex-linked differences in the post-6MWT values of forced expiratory volume in the first second A: forced expiratory volume in the first second for males (blue graph), B: forced expiratory volume in the first second for females (pink graph)

**Figure 4:**
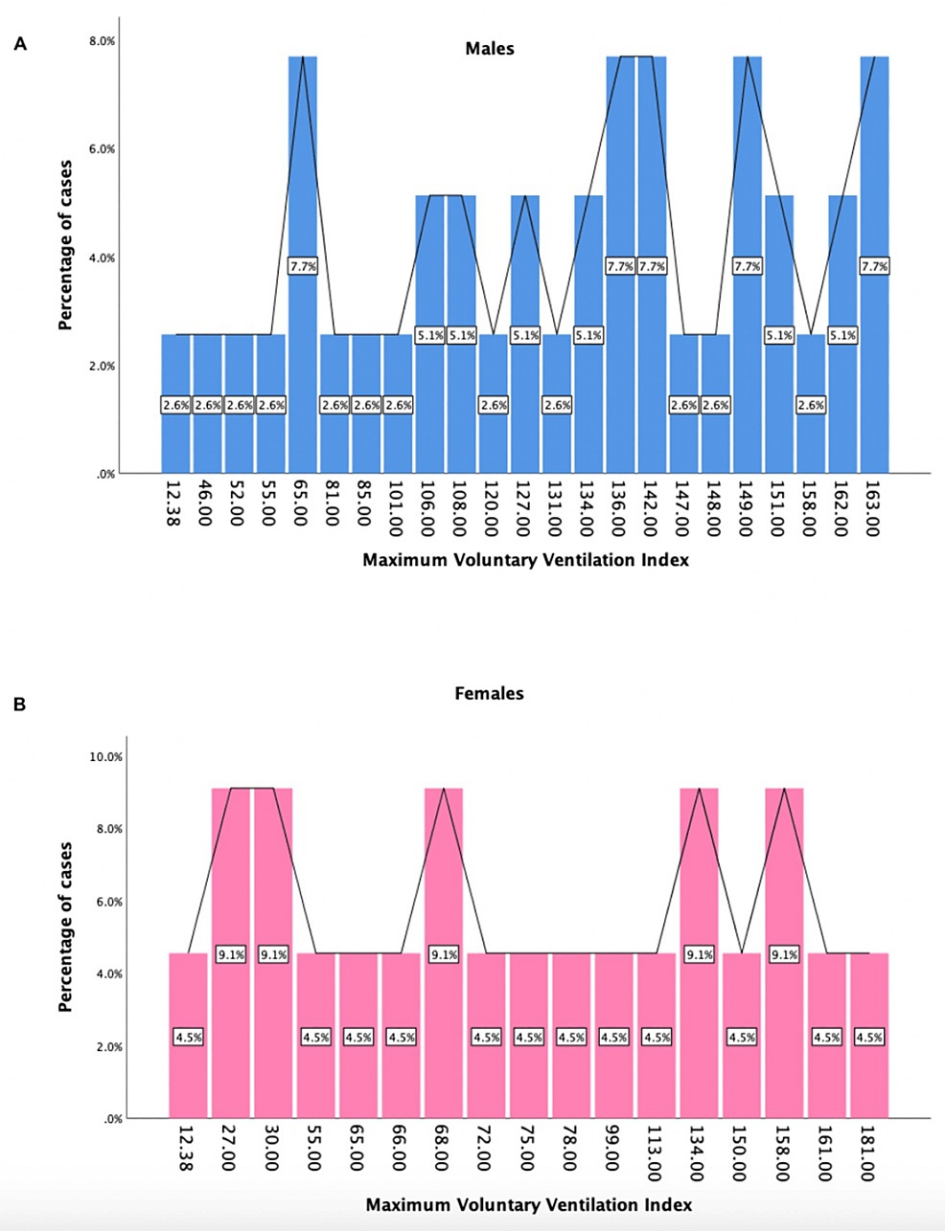
Sex-linked differences in the post-6MWT values of maximum voluntary ventilation index A: maximum voluntary ventilation index for males (blue graph), B: maximum voluntary ventilation index for females (pink graph)

Sex-linked differences in the post-6MWT significant pulmonary function test values (FEV1, FVC, and MVV index) can be seen in Figures 2-4.

Among males, the Pearson correlation analysis reveals that most of the PFT values are significantly correlated with each other, except for FEV%, which is only correlated with FEF50 and FEF75 (Table 4). The Pearson correlation for females shows that all PFT values are significantly correlated with each other, except for FEV% (Table 5).

**Table 4:** Pearson correlation for male subjects after a six-minute walk test SBP: systolic blood pressure, DBP: diastolic blood pressure, HR: heart rate, VC: vital capacity, FEV1: forced expiratory volume in the first second, FVC: forced vital capacity, FEV%: forced expiratory volume %, FEF25: forced expiratory flow 25, FEF50: forced expiratory flow 50, FEF75: forced expiratory flow 75, MVV IND: maximum ventilatory volume index, PEF: peak expiratory flow

Variables	SBP (mmHg)	DBP (mmHg)	HR (beats/minute)	VC	FEV 1	FVC	FEV%	FEF25	FEF50	FEF75	MVV IND	PEF
Oxygen saturation (%)	-0.125	-0.085	0.123	0.267	0.234	0.284	-0.350	-0.066	0.001	-0.060	0.127	-0.132
SBP (mmHg)	-	0.707	0.228	-0.230	-0.138	-0.074	-0.167	-0.223	-0.233	-0.377	0.017	-0.204
DBP (mmHg)	-	-	0.182	-0.288	-0.243	-0.170	-0.245	-0.381	-0.348	-0.459	-0.165	-0.383
HR (beats/minute)	-	-	-	0.106	-0.048	-0.032	-0.009	-0.156	0.116	-0.092	0.001	-0.101
VC	-	-	-	-	0.915	0.943	-0.310	0.664	0.508	0.242	0.829	0.752
FEV1	-	-	-	-	-	0.962	-0.100	0.832	0.698	0.375	0.939	0.846
FVC	-	-	-	-	-	-	-0.332	0.704	0.515	0.238	0.917	0.789
FEV%	-	-	-	-	-	-	-	0.265	0.505	0	-0.072	0.091
FEF25	-	-	-	-	-	-	-	-	0.806	0.475	0.811	0.942
FEF50	-	-	-	-	-	-	-	-	-	0.703	0.667	0.692
FEF75	-	-	-	-	-	-	-	-	-	-	0.404	0.409
MVV IND	-	-	-	-	-	-	-	-	-	-	-	0.846

**Table 5:** Pearson correlation for female subjects after a six-minute walk test SBP: systolic blood pressure, DBP: diastolic blood pressure, HR: heart rate, VC: vital capacity, FEV1: forced expiratory volume in the first second, FVC: forced vital capacity, FEV%: forced expiratory volume %, FEF25: forced expiratory flow 25, FEF50: forced expiratory flow 50, FEF75: forced expiratory flow 75, MVV IND: maximum ventilatory volume index, PEF: peak expiratory flow

Variables	SBP (mmHg)	DBP (mmHg)	HR (beats/minute)	VC	FEV1	FVC	FEV%	FEF25	FEF50	FEF75	MVV IND	PEF
Oxygen saturation (%)	-0.159	0.272	-0.629	0.036	0.196	0.265	0.067	-0.015	0.056	0.158	0.042	-0.017
SBP (mmHg)	-	0.505	0.381	-0.286	-0.449	-0.322	0.176	-0.532	-0.459	-0.291	-0.260	-0.506
DBP (mmHg)	-	-	0.439	-0.185	-0.099	-0.044	0.271	-0.098	-0.151	-0.233	0.068	-0.031
HR (beats/minute)	-	-	-	-0.041	-0.100	-0.163	0.171	0.093	0.085	-0.025	0.092	0.120
VC	-	-	-	-	0.926	0.930	-0.333	0.820	0.790	0.531	0.848	0.825
FEV1	-	-	-	-	-	0.955	-0.228	0.906	0.836	0.576	0.912	0.902
FVC	-	-	-	-	-	-	-0.399	0.792	0.714	0.540	0.860	0.797
FEV%	-	-	-	-	-	-	-	-0.086	0.011	-0.037	-0.132	-0.108
FEF25	-	-	-	-	-	-	-	-	0.910	0.567	0.913	0.988
FEF50	-	-	-	-	-	-	-	-	-	0.777	0.786	0.866
FEF75	-	-	-	-	-	-	-	-	-	-	0.473	0.507
MVV IND	-	-	-	-	-	-	-	-	-	-	-	0.921

Based on the test of significance, FEV1, FVC, and MVV index showed significant differences between the two genders. Hence, these three variables were considered as predictors, and three separate linear regression modeling analyses were conducted to drive three regression equations.

Based on the three methods of regression mentioned in the Materials and Methods section (stepwise, forward, and enter/remove), age, gender, oxygen saturation, DBP, HR, FEF75, height, and weight were commonly excluded. The analysis showed that model 1 (with all the variables) had an R2 value of 0.983 with a p-value of 0.001. Model 2 (with the excluded variables) showed an R2 value of 0.000 with a p-value of 0.896. This shows that removing these variables had a minimal impact on predicting FVC. Based on analysis, the value of FVC would be 3.44% higher in males compared to females. The linear regression equation obtained for predicting FVC is as follows: FVC = -13.550.121 × SBP 0.062 × VC + 0.884 × FVC + 0.275 × FEV% + 0.216 × FEF25 + 0.082 × FEF50 + 0.058 × MVV - 0.147 × PEF.

The three methods of regression, namely, stepwise, forward, and enter/remove, were used for analysis. The following variables were not included in the final analysis: age, gender, oxygen saturation, SBP, DBP, HR, VC, FEF75, height, and weight. The analysis showed that model 1, which included all the variables, had an R2 value of 0.994 and a p-value of 0.000. Model 2 showed an R2 value of 0.002 with a p-value of 0.415. This shows that removing these variables had a minimal impact on the adjusted R2 value of model 1. According to statistical analysis, the predicted value of FVC would be 0.165% higher in males compared to females. The linear regression equation obtained for predicting FEV1 is as follows: FEV1 = 26.494 + 0.948 × FVC + 0.248 × FEV% + 0.198 × FEF25 + 0.074 × FEF50 - 0.067 × MVV - 0.098 × PEF.

Linear regression analysis was performed using the stepwise, forward, and enter/remove methods. The following variables were excluded from the final analysis: gender, oxygen saturation, DBP, HR, VC, FEV%, FEF25, FEF50, FEF75, FVC, and weight. The analysis showed that model 1, which included all the variables, had an R2 value of 0.932 with a p-value of 0.000. Model 2 showed an R2 value of 0.015 with a p-value of 0.132. This shows that removing these variables had a minimal impact on predicting the MVV index. According to the analysis, the MVV index value would be 14.6% lower in females than in males. The linear regression equation obtained for predicting the MVV index is as follows: MVV index = - 9.47 - 0.6876 × age + 0.717 × SBP + 0.88 × FEV1 + 0.485 × PEF - 0.607 × height.

## Discussion

To our knowledge, the present study is the first of its kind to comprehensively evaluate sex-related differences in lung function following 6MWT. We found that on comparison of means of various parameters between post-COVID-19 males and females after performing the six-minute walk test, there was a significant difference between the following PFTs: FEV1 (p = 0.038), FVC (p = 0.041), and MVV index (p = 0.011) (Table 1). Sex-linked differences in the post-6MWT significant pulmonary function test values (FEV1, FVC, and MVV index) can be seen in Figures 2-4.

To date, there has been no reported evidence of a significant relationship between COVID-19 and PFTs in our population. These significant parameters are of utmost importance, given the study’s sex-based design. FEV1 for males was 120.61 ± 28.05, while for females, it was 103.50 ± 33.91, resulting in a mean difference of -17.11 (p = 0.038). FEV1 is described as more effort-dependent compared to FEF25-FEF75 [18]. FEV1 represents the expiratory flow over the first second of the entire expiratory process and is a measurement of dynamic volume most often used in conjunction with the FVC in spirometry analysis [19]. The measurement reflects an early, effort-dependent portion of the curve, making it sensitive and reproducible [20]. The endpoint of spirometry is clearly defined, making the calculations between measured and reference values more reliable [21].

Our study found that the FVC for males was 114.87 ± 27.69, while for females, it was 98.72 ± 31.14, resulting in a mean difference of -16.14 (p = 0.041). Baratto et al. state that there is a moderate reduction in forced vital capacity (79 ± 40%) post-COVID-19. This reduced capacity of the body to respond to exercise could be due to persistent pulmonary parenchymal pathology or pulmonary vascular disruption, including resulting anemia due to impaired peripheral extraction of iron after COVID-19 [22]. Our study reports that the MVV index for males was 119.93 ± 39.09, while for females, it was 89.15 ± 51.58, resulting in a mean difference of -30.77 (p = 0.011). MVV is the maximum volume of air that a person can exhale from their lungs in one minute using voluntary effort. This respiratory parameter is particularly useful in specific situations, especially when assessing stress tolerance [23,24]. Frija-Masson et al. conducted a study where they assessed the PFTs one month after a COVID-19 infection. The research study revealed that one month after recovering from COVID-19, the majority of patients experienced a mild alteration in lung function, including both restrictive and obstructive types [14].

Torres-Castro et al. conducted a systematic review of studies that examined the impact of COVID-19 on PFTs in post-COVID-19 patients. They discovered that 7% of the patients exhibited an obstructive pattern of disease, as indicated by deranged FEV1/FVC ratio [25]. Another review conducted by van den Borst et al. revealed that 90% of patients displayed signs of residual pulmonary parenchymal abnormalities, as evidenced by PFTs and diffusion capacity values. However, they only considered post-COVID-19 severe and critically ill patients, which could explain the high percentage of persistent lung fibrosis and parenchymal derangement [26]. Several studies have checked PFT values in COVID-19 patients, but none have reported sex-related differences in PFTs following physiological stress. Thus, our study is unique and meaningful and adds significant value to the medical literature [1,27].

Our study reports the mean elapsed time (post-COVID-19) (7.21 ± 3 months). All cases exhibited symptoms of COVID-19 illness. Fever (86.9%), dry cough (73.8%), and body aches (90.2%) were reported as the top three symptoms. A study found fever, fatigue, and cough to be the most common COVID-19 symptoms, documented in adult patients who did not require hospitalization [28]. Loss of taste and loss of smell sensation are reported more frequently in COVID-19 than in any other respiratory viral illness [29]. It was noted that fever and dry cough were the predominant symptoms in over 90% of hospitalized patients [30].

Strengths and limitations

One of the key strengths of this research is that it compared gender differences in PFTs after stress application post-COVID-19. The majority of confounders were taken into account, such as the general health status, past medical history, and body mass index (BMI) of the patients. The biggest confounding factor could be the participants’ medical history of respiratory tract diseases, such as asthma, which was addressed during the recruitment stage. This crucial exclusion criterion was overlooked by many of the previously conducted research studies. One of the limitations is that the waist circumference (as well as abdominal obesity) of the patients was not quantified. This is important because A Body Shape Index (ABSI) is a factor that affects PFTs. This, along with a smaller and less diverse sample size consisting mainly of young adults, are the limitations of the study.

Future work

In the future, research should involve more advanced parameters, such as ABSI, and consider important components such as metabolic syndrome and, in turn, abdominal obesity. The measurement of the diffusion capacity of the lungs should be included in addition to other spirometry parameters to obtain more reliable results. Furthermore, blood biomarkers that chronically affect PFTs can also be researched and statistically compared to provide further details about the pathophysiology.

## Conclusions

Among the PFTs, FEV1, FVC, and MVV index showed significant differences between the two genders. Furthermore, all of the PFTs were significantly correlated with each other, except for FEV%. To conclude, the results showed that there are gender-based differences in PFTs among the participants who had COVID-19 infection. The values of significant post-stress variables, namely, FEV1 (p = 0.038), FVC (p = 0.041), and MVV index (p = 0.011), were higher for males compared to females. A gender-based difference also exists between SBP and DBP in males and females, with males having higher SBP and DBP. Recognizing these differences might be crucial for accurate diagnosis and the management of post-COVID-19 patients during the follow-up period.

## Figures and Tables

**Figure 5:**
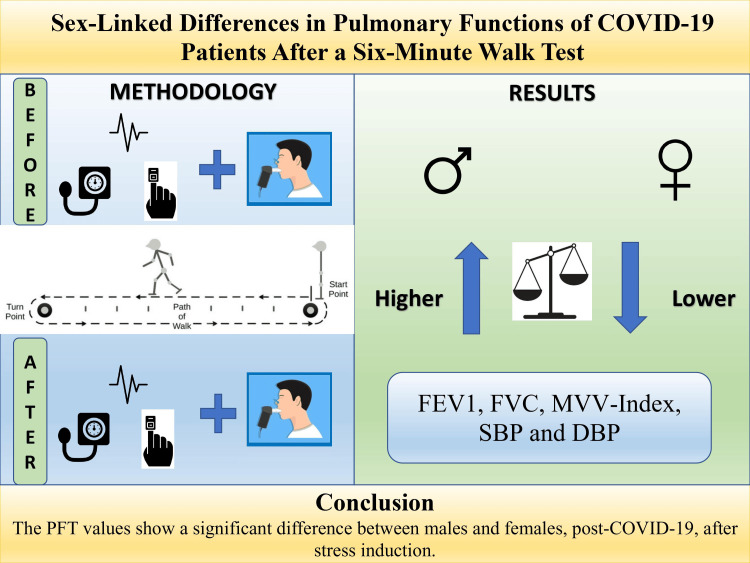
Graphical abstract ♂: male gender, ♀: female gender, PFT: pulmonary function test, COVID-19: coronavirus disease 2019, SBP: systolic blood pressure, DBP: diastolic blood pressure, FEV1: forced expiratory volume in the first second, FVC: forced vital capacity, MVV: maximum ventilatory volume

## Appendices

Figure 5 shows the graphical abstract of the study. 
